# Recent advances of nanotechnology-based tumor vessel-targeting strategies

**DOI:** 10.1186/s12951-021-01190-y

**Published:** 2021-12-20

**Authors:** Dongjie Zhu, Yang Li, Zhengjia Zhang, Zeyu Xue, Zhenglai Hua, Xinyi Luo, Ting Zhao, Cheng Lu, Yuanyan Liu

**Affiliations:** 1 School of Chinese Materia Medica, Beijing University of Chinese Medicine, Beijing, 100029 China; 2 Institute of Basic Research in Clinical Medicine, China Academy of Chinese Medical Sciences, Beijing, 100700 China

**Keywords:** Nanomedicines, Tumor vessel-targeting strategies, Anti-tumor therapy, Combined therapy

## Abstract

Tumor vessels can provide oxygen and nutrition for solid tumor tissue, create abnormal tumor microenvironment (TME), and play a vital role in the development, immune escape, metastasis and drug resistance of tumor. Tumor vessel-targeting therapy has become an important and promising direction in anti-tumor therapy, with the development of five anti-tumor therapeutic strategies, including vascular disruption, anti-angiogenesis, vascular blockade, vascular normalization and breaking immunosuppressive TME. However, the insufficient drug accumulation and severe side effects of vessel-targeting drugs limit their development in clinical application. Nanotechnology offers an excellent platform with flexible modified surface that can precisely deliver diverse cargoes, optimize efficacy, reduce side effects, and realize the combined therapy. Various nanomedicines (NMs) have been developed to target abnormal tumor vessels and specific TME to achieve more efficient vessel-targeting therapy. The article reviews tumor vascular abnormalities and the resulting abnormal microenvironment, the application of NMs in the tumor vessel-targeting strategies, and how NMs can improve these strategies and achieve multi-strategies combination to maximize anti-tumor effects.

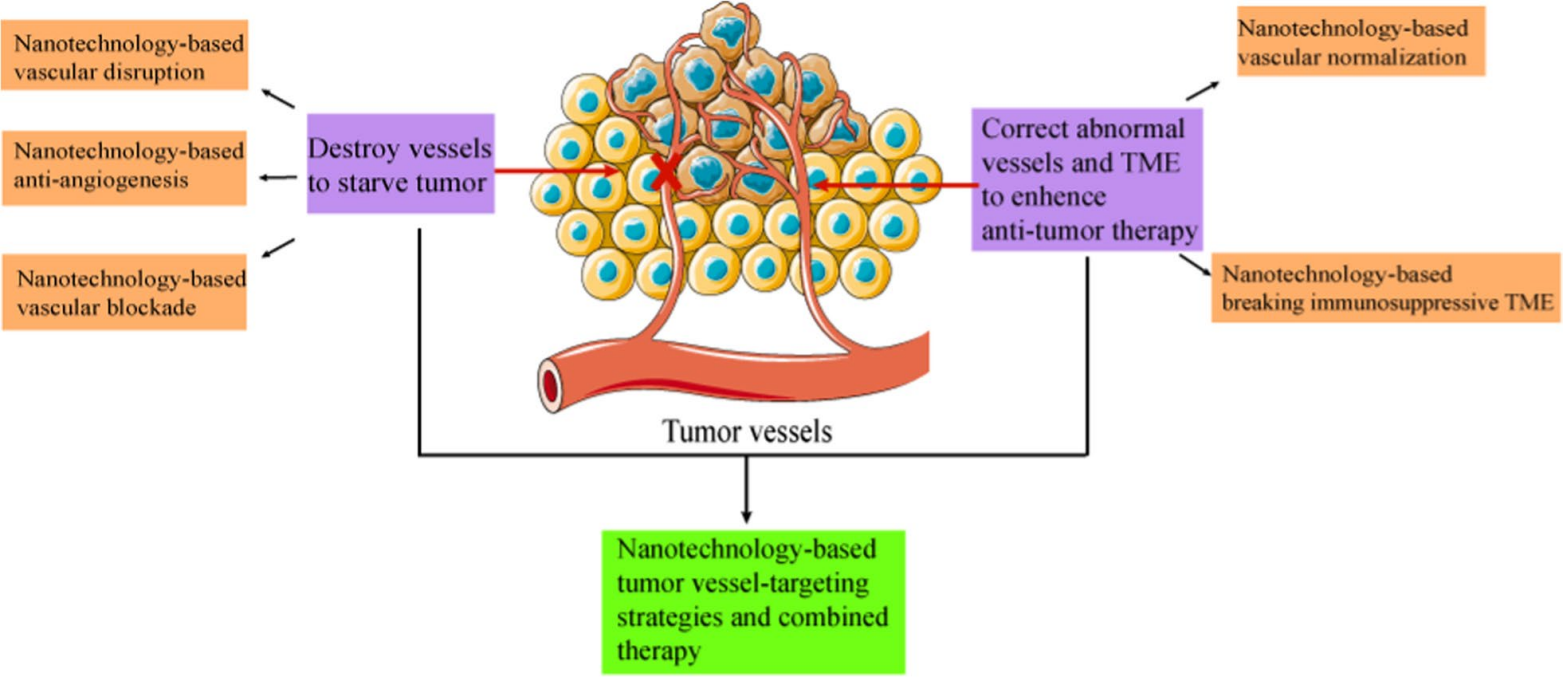

## Introduction

Tumor vessels are important for solid tumor growth and development, which are mainly composed of newly formed vessels. Tumors need blood vessels to obtain nutrients and oxygen, as well as to remove carbon dioxide and metabolic waste. When tumor tissue is larger than 2 mm^3^, it must generate blood vessels to obtain the nutrients for its survival and development [1]. Remarkably, tumor vessels are chaotic, tortuous and incomplete in structure and are locally leaky and compressed, resulting in poor perfusion, hypoxic, acidic and immunosuppressive TME, increased interstitial fluid pressure (IFP) and favorable conditions for tumor metastasis. Poor perfusion and increased IFP limit the delivery and penetration of chemotherapeutic drugs into tumor tissue, while the abnormal TME limits the efficacy of chemotherapy, radiotherapy, and immunotherapy. Therefore, abnormal tumor vessels are recognized as an important culprit of failure in tumor therapy [2]. The metastasis and proliferation of cancer cells through leaky blood vessels (hematogenous diffusion) is also an important pathway in the metastatic cascade [3].

In recent years, in view of the deteriorated role and abnormal characteristics of tumor vessels, two types of vessel-targeting strategies have been proposed according to different therapeutic concepts. One is to inhibit tumor growth by destroying tumor vascular function and cutting off the transport of nutrients and oxygen for tumor tissue [4–6]. The other is to enhance chemotherapy, radiotherapy and immunotherapy by correcting tumor vascular abnormalities and restoring normal function [2, 7]. Vascular disruption strategy aims at disrupting existing endothelial cells (ECs) of tumor vessels, incurring thrombosis and blocking blood supply to the tumor [4, 8]. Anti-angiogenic strategy aims at inhibiting tumor angiogenesis via blocking pro-angiogenic pathways (e.g. vascular endothelial growth factor (VEGF) pathway and angiopoietin 2(Ang 2) pathway) [5]. Vascular blockade strategy refers to blocking tumor vessels by triggering coagulation reaction or gel phase transformation [6]. Vascular normalization strategy is to correct the abnormal state of tumor vessels, restore the normal function of tumor vessels, increase perfusion and oxygen supply, and improve the delivery and efficacy of chemotherapeutic drugs [2, 9]. Meanwhile, targeting tumor vessels to break the immunosuppressive TME caused by tumor vascular abnormality and enhance the infiltration of anti-tumor immune cells, thus enhancing immunotherapy is also a therapeutic strategy [7]. However, to some extent, single vessel-targeting strategy also has certain defects for the poor pharmacokinetics, insufficient tumor accumulation and retention, and even severe toxicity with non-specificity of drugs. For instance, vascular disrupting agents (VDAs) usually have large distribution volumes, shorter half-life and cardiotoxicity after systemic administration, which limit their therapeutic efficacy [4]. Single vascular disruption strategy and vascular blockade strategy are often associated with marginal tumor survival and recurrence, because the peripheral vessels of tumors are more mature and abundant, and the external tumor cells can obtain nutrients from nearby normal tissues. Anti-VEGF therapy alone is usually associated with drug resistance due to the diversity and complexity of tumor angiogenesis pathways. Meanwhile, the aggressiveness of tumor will be enhanced due to the increased level of hypoxia in tumor tissues [4, 5, 8, 10]. Therefore, innovative approaches to improve tumor vessel-targeting strategies and achieve combined therapy are urgently needed.

**Fig. 1:**
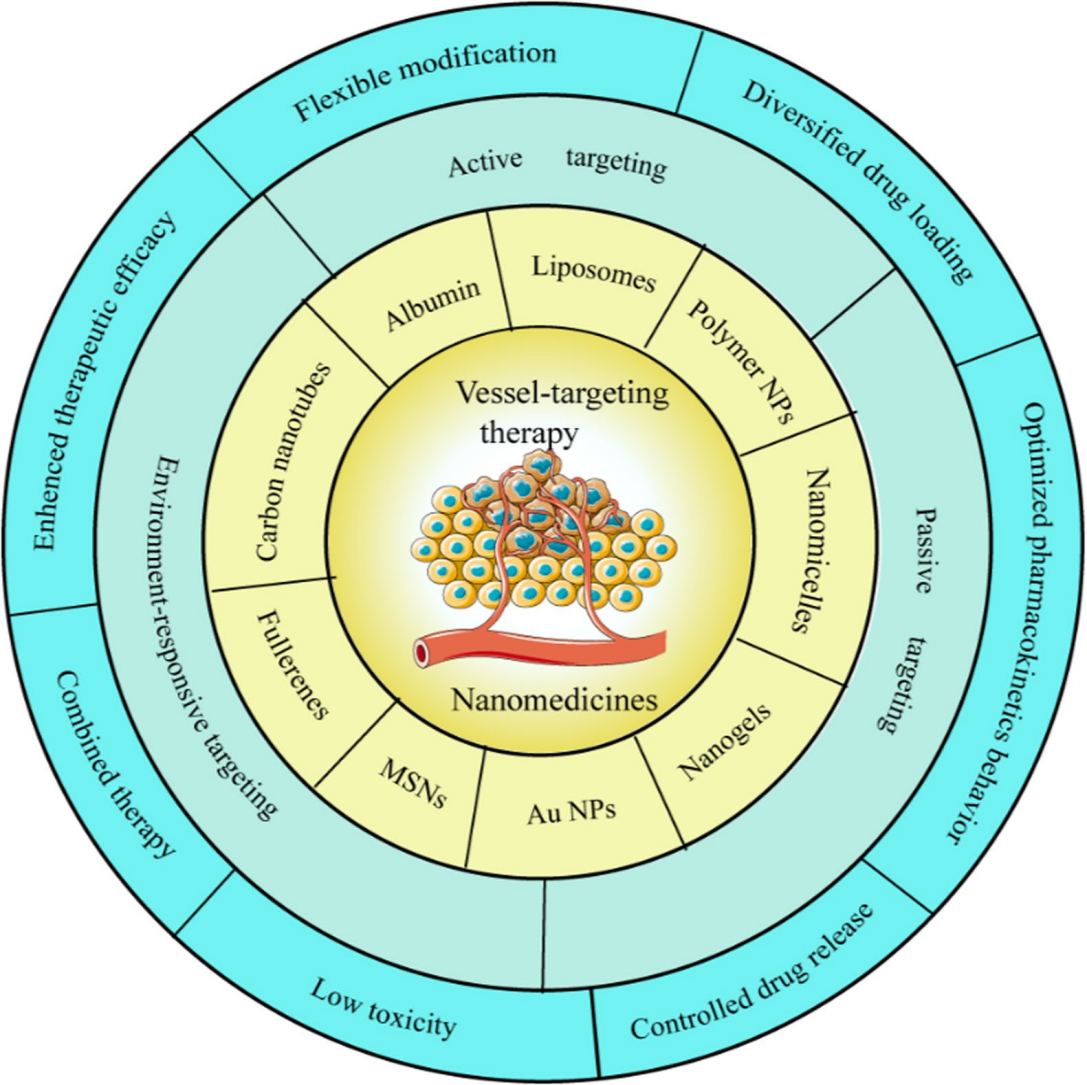
Schematic diagram of various nanocarriers and targeting methods that can be used for tumor vessel-targeting strategies and the advantage of NMs

Nanotherapeutics have shown attractive properties in improving the efficacy of drug therapy or revolutionizing current vessel-targeting strategies. The most common nanomaterials used for vessel-targeting therapy include natural or synthetic organic nanomaterials, such as albumins, liposomes, polymer nanoparticles (NPs), nanomicelles and nanogels; inorganic or metallic NPs, such as gold NPs (Au NPs), mesoporous silica NPs (MSNs); and carbon-based materials, such as fullerenes and carbon nanotubes. Nanocarriers can load vessel-targeting drugs inside or on their surfaces through hydrophobic effect, electrostatic interaction, or chemical bonding to form NMs, so as to achieve targeting delivery and enhanced efficacy. In addition, the physicochemical properties of nanocarriers, such as surface charge, size and stimulus responsiveness can be easily controlled during the manufacturing process, facilitating more efficient and combined drug delivery. The design of multi-functional and biocompatible nanocarriers enable several desirable properties, such as delivering drugs to specific tissue or cells, triggering sustained drug release in particular locations and co-delivering multiple drugs [5, 8, 11]. Leaky tumor vessels also are important contributors to the enhanced permeability and retention (EPR) effect, which can be used to promote passive accumulation of NMs in tumor tissue. By modifying with specifically targeting ligand, such as peptides, antibodies, and aptamers, NMs can accurately and actively target tumor vessels to reduce adverse reactions and off-target effects, and improve efficacy. In addition, some TME-responsive nanomaterials have also been developed for controlled and selective drug release in response of TME formed specific pH, temperature and enzymes (Fig. 1) [5, 11].

In the review, we summary the application of NMs in tumor vessel-targeting strategies described above, and how NMs can improve these vessel-targeting strategies and achieve multi-strategy combination. The article also systematically discusses the challenges and prospects of nanotechnology-based tumor vessel-targeting strategies, which may be a booming angle for tumor therapy.

## The abnormal properties of tumor vessels

### Tumor vessels are structurally and functionally abnormal

**Fig. 2:**
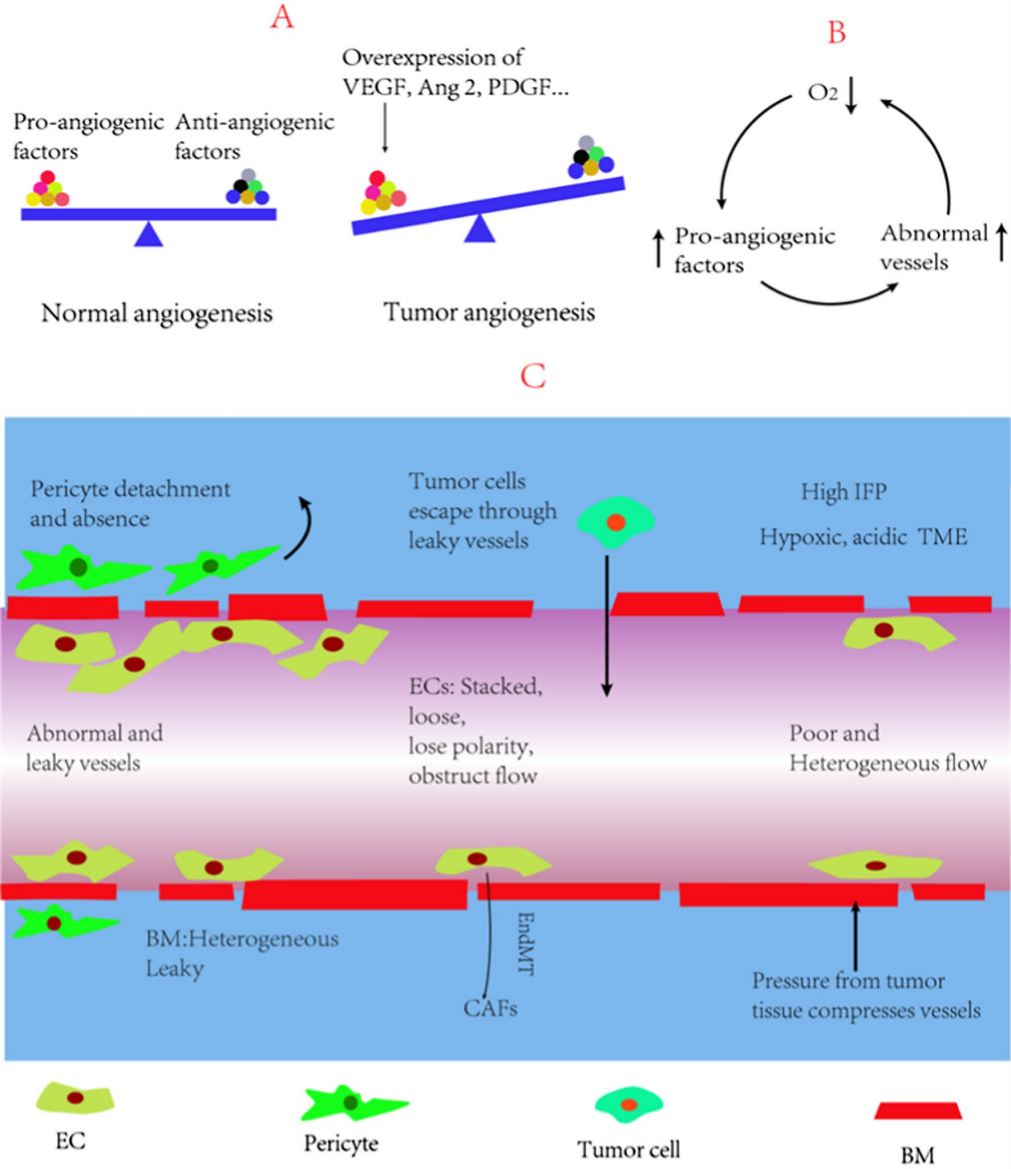
Tumor angiogenesis and vascular structure and function are abnormal. **A** Normally, a delicate balance between pro-angiogenic factors and anti-angiogenic factors is maintained to strictly closely regulate angiogenesis. But tumor tissue continued to overexpress pro-angiogenic factors, which kept tumor tissue in a pro-angiogenic state. **B** Hypoxia promotes the overexpression of pro-angiogenic factors in tumors, which will lead to tumor vascular abnormalities, which in turn will reduce oxygen delivery, forming a vicious cycle. **C** The structure and function of tumor vascular ECs and vascular walls are abnormal, leading to an abnormal TME, and promoting the development and metastasis of tumor. *EC* endothelial cell, *BM* basement membrane, *CAF* cancer-associated fibroblast, *EndMT* endothelial-mesenchymal transition

Most tumor neovascularization seems to occur via angiogenesis, which refers to the process of generating new blood vessels from existing ones. Angiogenesis is a complex physiological process, and in normal conditions, a delicate balance between pro-angiogenic factors and anti-angiogenic factors is maintained to strictly regulate angiogenesis and maintain homeostasis [1, 12]. Because of the vigorous growth and proliferation, tumor tissue needs to consume a lot of oxygen and nutrients, and are always in a state of hypoxia. Tumor tissue maintains a continuous pro-angiogenic state by increasing the expression of a series of pro-angiogenic factors, such as VEGF, Ang 2, epidermal growth factor (EGF) and platelet-derived growth factor (PDGF), to fulfill their increased demand for nutrients and oxygen (Fig. 2A) [12, 13]. Although the overexpressed pro-angiogenic factors can promote angiogenesis, the newly formed vessels are extremely immature and structurally and functionally abnormal, which cannot achieve the purpose of oxygen and energy supply (Fig. 2C) [2, 12].

Tumor vessels have a chaotic, tortuous and incomplete structure. The vascular basement membrane (BM) plays a role in maintaining the proper placement of endothelial cells (ECs) and pericytes. The tumor vascular BM has heterogeneous thickness and holes, and is loosely connected to ECs and pericytes. Tumor vascular ECs are also different from normal ECs. Typically, the normal ECs are regularly arranged, polarized along blood flow and closely connected to each other. Instead, tumor ECs are activated, lose polarity, overlap with each other, and are loosely connected to BM [2, 14]. The connections between tumor ECs are also loose, while in some sites, ECs occur endothelial-mesenchymal transition (EndMT) or die and leave behind gaps. The vessels become leakier and have high permeability. EndMT is an important source of cancer-associated fibroblasts (CAFs), which promote development and metastasis of tumors [15]. Pericytes can support the vascular structure and maintain vascular integrity. During angiogenesis, pericytes need to detach from ECs. Tumor vessels have less pericyte coverage due to the overexpression of VEGF and Ang 2. Moreover, pericytes are loosely connected to ECs and less contractile, thus losing the ability to regulate blood flow and vascular permeability [2, 14].

The abnormal structure naturally leads to abnormal function. The ability of tumor vessels to transport nutrients and to clear metabolic waste via the lymphatic system is severely impaired. The irregular and loosened ECs and structurally incomplete vessel wall contribute to vessel leakiness. Leakiness could reduce blood flow by increasing blood viscosity and hematocrit, which causes resistance to flow. Moreover, leakiness could also reduce the pressure gradient in vessels, which is a key factor in driving blood flow. Tumor vessels have higher permeability than normal vessels, resulting in increased accumulation of vascular macromolecules in tumor tissues and higher interstitial fluid pressure (IFP). In addition, due to the increased pressure from tumor tissue, some structurally incomplete tumor vessels are compressed into a lack of flow. The pressure and leakiness together lead to spatial and temporal heterogeneities of tumor perfusion [12, 14].

Tumors crave for oxygen, which up-regulate the levels of pro-angiogenic factors to promote angiogenesis. Paradoxically, the overexpression of pro-angiogenic factors result in tumor vascular abnormalities, which in turn impairs perfusion and oxygen delivery, continuing the vicious cycle (Fig. 1B) [16].

### Abnormal tumor vessels contribute to immunosuppressive TME

**Fig. 3:**
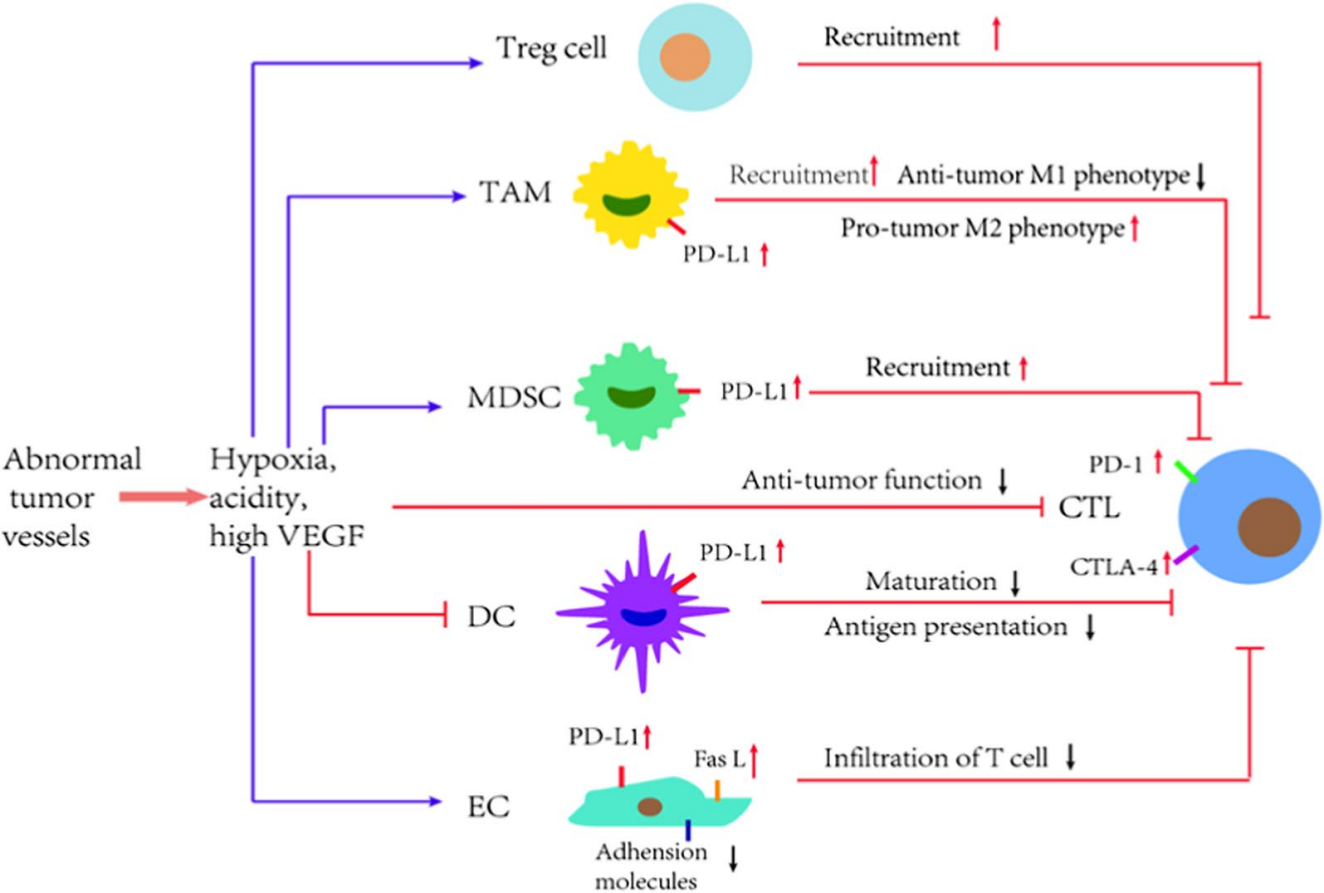
The abnormal tumor vessels lead to immunosuppression in TME. Abnormal tumor vessels contribute to a hypoxic, acidic TME, as well as high levels of pro-angiogenic factors such as VEGF, which result in immunosuppression. These mechanisms include increasing the accumulation of immunosuppressive Treg cells and MDSCs, promoting the accumulation of TAMs and the polarization towards the pro-tumor M2 phenotype, inhibiting DCs maturation, leading to impaired antigen presentation, inhibiting CTLs activation. Hypoxia can also directly limit CTLs function. Tumor vascular ECs also exhibit immunosuppressive phenotypes, reducing the infiltration of CTL into tumor tissue. In addition, inhibitory immune checkpoint pathways are generally activated in TME to limit the anti-tumor function of CTLs. PD-L1 expression was up-regulated in TAMs, DCs, ECs, and tumor-infiltrating CTLs usually up-regulated PD-1 and other inhibitory immune checkpoint receptors, indicating their dysfunction or failure and limiting their killing ability to tumor cells. *CTL* cytotoxic T lymphocyte, *Treg cell* regulatory T cell, *DC* dendritic cells, *MDSCs* myeloid-derived suppressor cells, *TAM* tumor-associated macrophage, *PD-1* programmed cell death 1, *PD-L1* programmed cell death ligand 1, *FasL* Fas ligand, *CTLA-4* cytotoxic T-lymphocyte-associated protein 4

Studies have shown that the non-productive and abnormal tumor vessels are major influencers of the immunosuppressive TME (Fig. 3) [17]. Tumor vascular ECs can reduce the recruitment and activation of cytotoxic T lymphocytes (CTLs) by expressing a variety of immunosuppressive molecules, such as programmed cell death 1 ligand 1/2 (PD-L1/2), Fas ligand (FasL). FasL is a death ligand for activated T cells, which can selectively induce CTLs apoptosis but not kill regulatory T cells (Tregs). Meanwhile, tumor vascular ECs also can secrete immunosuppressive cytokines (e.g. interleukin 10 (IL-10), prostaglandin E2 (PGE2) and VEGF). CTLs lose anti-tumor function or apoptosis before they can cross tumor vessels and enter TME [18–20]. In addition, tumor vascular ECs also downregulates the level of adhesion molecules on cell surface, which limits the trafficking of immune effector cells into tumors, such as vascular cell adhesion molecule-1 (VCAM-1) and intercellular adhesion molecule-1 (ICAM-1) [18].

Leakiness and compression of tumor vessels contribute to hypoxia and acidity of TME, which promote immunosuppression. Hypoxia can polarize tumor-associated macrophages (TAMs) to immunosuppressive phenotype (M2-TAMs), thereby impairing the recruitment and activation of effector lymphocytes [21]. Hypoxia can also cause changes in metabolic pathways of tumor cells, resulting in accumulation of immunosuppressive metabolites (e.g., adenosine). Hypoxia can also directly inhibit the function of T-effector cell [22]. Moreover, hypoxia can directly induce the upregulation of inhibitory immune checkpoints ligand (e.g., PD-L1 and cytotoxic T-lymphocyte-associated protein 4, CTLA-4) on dendritic cells (DCs), myeloid-derived suppressor cells (MDSCs), TAMs and tumor cells through HIF-1α [23].

Some pro-angiogenic factors, such as VEGF, are also immunosuppressive molecules. VEGF signaling promotes immunosuppression through many mechanisms. First, VEGF induces vascular leakiness and causes hypoxia, which is an important cause of immunosuppression [22]. Second, VEGF increases the recruitment of immunosuppressive cells, such as Treg cells, MDSCs, and M2-TAMs [24, 25]. Third, VEGF inhibits maturation of DCs and antigen presentation, which impedes activation and function of T cells [26]. Fourth, VEGF blocks CTLs trafficking and mediates CTLs exhaustion and anergy by modulating the inhibitory immune checkpoints receptor on T cells, including PD-1, CTLA-4, and Tim-3 [25, 27].

The abnormal tumor vessels are essential for tumor growth, development, immune escape and metastasis, and are also a key reason for the failure of anti-tumor therapy. Poor perfusion and high IFP caused by abnormal tumor vessels reduce drug delivery to tumor tissue. The hypoxic, acidic, and immunosuppressive TME can reduce the therapeutic sensitivity on radiotherapy, chemotherapy, and immunotherapy, and can also stimulate pre-malignant reprogramming of tumor cell metabolism and promote metastasis through leaky vessels [2]. Therefore, using NMs to target tumor vessels for anti-tumor therapy or enhance the efficacy of other therapies will open new horizons for tumor therapy.

## NMs-mediated disruption of tumor vessels

The solid tumor growth is always accompanied by angiogenesis, which in turn promotes growth. Solid tumors have an abundant vascular distribution in the periphery, while an insufficient vascular distribution in the interior [4, 28]. Therefore, disrupting tumor vessels can lead to extensive central necrosis. VDAs are specific vessel-targeting drugs that can specifically disrupt the immature vascular ECs, thereby triggering thrombosis [4]. NMs loaded with VDAs or some special nanomaterials can selectively disrupt tumor vessels to inhibit tumor growth.

### NMs loaded with VDAs

VDAs belong to small molecule drugs, usually have large distribution volumes and shorter half-life after systemic administration, which limit their therapeutic efficacy [28]. NMs can effectively accumulate VDAs in tumor sites, continuously release, prolong half-life, and enhance the activity of disrupting tumor vessels.

Combretastatin A4 (CA4) is a tubulin-binding agent VDAs. After binding, rapid microtubule depolymerization occurs, resulting in rapid changes in ECs shape, leading to vascular disruption. Liu [29] derived a poly (L-glutamic acid)-CA4 conjugate NPs to enhance the efficacy of CA4 in tumor therapy. The NMs could increase the tumor retention and accumulation of CA4 via EPR effect, and maintain a high level of CA4 in tumor, which led to a prolonged time of disruption and markedly improved therapeutic effect. Liu [30] designed a GSH-responsive self-assembled nanocarrier, PEGylated poly (α-lipoic acid) copolymer, which loads CA4 through covalently linking and selectively delivered it to tumor tissues via environmental response. The nanocarrier had good blood circulation, high sensitivity to GSH stimulation, good stability under normal environment and good degradation ability under the action of GSH. The NMs can also achieve active targeting delivery of CA4 to tumor tissue by modifying targeting ligands. Hao [31] developed tumor targeting peptide APRPG modified monomethoxy poly (ethylene glycol)-poly (D, L-lactide) (mPEG-PLA) self-assembled nanomicelles loaded with CA4 via hydrophobic effect. The APRPG could specifically target the integrin αvβ3 in tumor angiogenesis sites. The NMs could markedly enhance the accumulation, sustained release and cellular uptake of CA4 in tumor tissue.

### Combination of NMs-loaded VDAs with other therapies

Although VDAs can effectively induce severe necrosis in the central region of tumors lacking blood vessels, it is inevitable that some tumor cells will survive in the periphery of tumors with high blood vessel density. One possible reason for this is that the residual external tumor cells can get oxygen and nutrients from the normal vessels in surrounding tissue, which generally are not affected by VDAs. These residual marginal cells can act as an important culprit of tumor metastasis and recurrence [4, 28, 32]. Therefore, VADs combined with chemotherapy, photothermal therapy (PDT) and other treatment regimens through NMs may achieve better anti-tumor effect.

Lv [28] prepared a polypeptide-based NPs that passively target tumor tissue, containing both VADs and chemotherapeutic agents. The methoxy PEG-b-poly-[(N-2-hydroxyethyl)-aspartamide] (mPEG-b-PHEA) was synthesized and covalently conjugated with 5,6-dimethylxanthenone-4-acetic acid (DMXAA), a flavonoid VDA. Then utilized mPEG-b-PHEA-DMXAA conjugate (PPD) for the encapsulation of doxorubicin (DOX) via hydrophobic and aromatic interaction. The tumor inhibition rate of the NMs was as high as 94.6%, which was higher than that of free drugs. Due to the EPR effect and high IFP, the NMs mainly accumulated in the tumor periphery. The continued release of DMXAA disrupted tumor vessels, effectively induced severe necrosis inside tumors, while the release of DOX killed the residual cells in the tumor periphery.

Li [33] designed an active targeting co-delivery nanosystem, using mesoporous silica NPs (MSNs) as the carrier, simultaneously loading CA4 and DOX, and the targeting peptides, iRGD, were modified on the surface. Because of the hydrogen bonding and electrostatic interaction between mesopores and DOX, the NPs could achieve a differentiated drug release. The fast release of the CA4 in tumor vessels had a synergetic effect with the slow release of DOX in tumor tissue. Only low dose DOX was needed, the NMs displayed a high-efficiency inhibition of tumor growth for a long time.

NMs can also realize the combined treatment of VDAs with PDT. Liang [34] designed an injectable nanocomposite hydrogel, which contained both PDT nanoagent-Prussian blue (PB)and VDAs-CA4. The nanoplatform achieved an “attack + guard” antitumor strategy, in which CA4 inhibit growth of tumor by blocking nutrients and oxygen supplies, while PB- mediated NIR irradiation could strongly attack most tumor cells. The hydrogel has good photothermal stability, high photothermal effect, injectability, excellent biocompatibility and the ability to continuously release CA4, which ensure the excellent efficacy of synergistic therapy.

### Targeted modification of nanomaterials disrupt tumor vessels

In addition to VDAs-induced tumor vascular disruption, some nanomaterials can also exert vascular disruption through radiotherapy or PDT.

Gold NPs (AuNPs) have good application potential as radiosensitizing agents. Kunjachan [35] prepared PEGylated AuNPs and co-modified with AF647, a near-infrared dye for imaging, and RGD, a tumor vascular targeting peptide. The NPs improved radiation outcome by actively targeting tumor vessels and image-guided irradiation to cause vascular damage. This dual-targeting NPs could improve radiotherapy by reducing the radiation dose needed to achieve the same therapeutic outcome and reducing side effects, or by increasing tumor damage. Fullerene NPs also disrupt tumor vessels through PDT. Guan [36] prepared photo-triggered NPs, β-alanine modified gadofullerene NPs, which could induce disruption of malignant tumor vessels under light illumination, and the oxygen in tumor vessels could be effectively converted into reactive oxygen species (ROS). The ROS could effectively kill cancer cells and destroy vascular ECs, resulting in the loss of intercellular connections and disruption of tumor vessels. Moreover, this NPs mediated PDT could activate the anti-tumor immune response and inhibit tumor metastasis.

## Anti-angiogenic therapy with NMs

Given the importance of angiogenesis for tumors, angiogenesis inhibitors have been shown to serve as novel anti-tumor drugs. Anti-angiogenic therapy based on nanotechnology is a potential new method. The main advantage of nano-drug delivery systems over anti-angiogenic agents alone is the ability to deliver anti-angiogenic drugs specifically to tumor angiogenesis sites while minimizing systemic toxicity [5, 37].

### NMs target on the VEGF signaling pathways

**Fig. 4:**
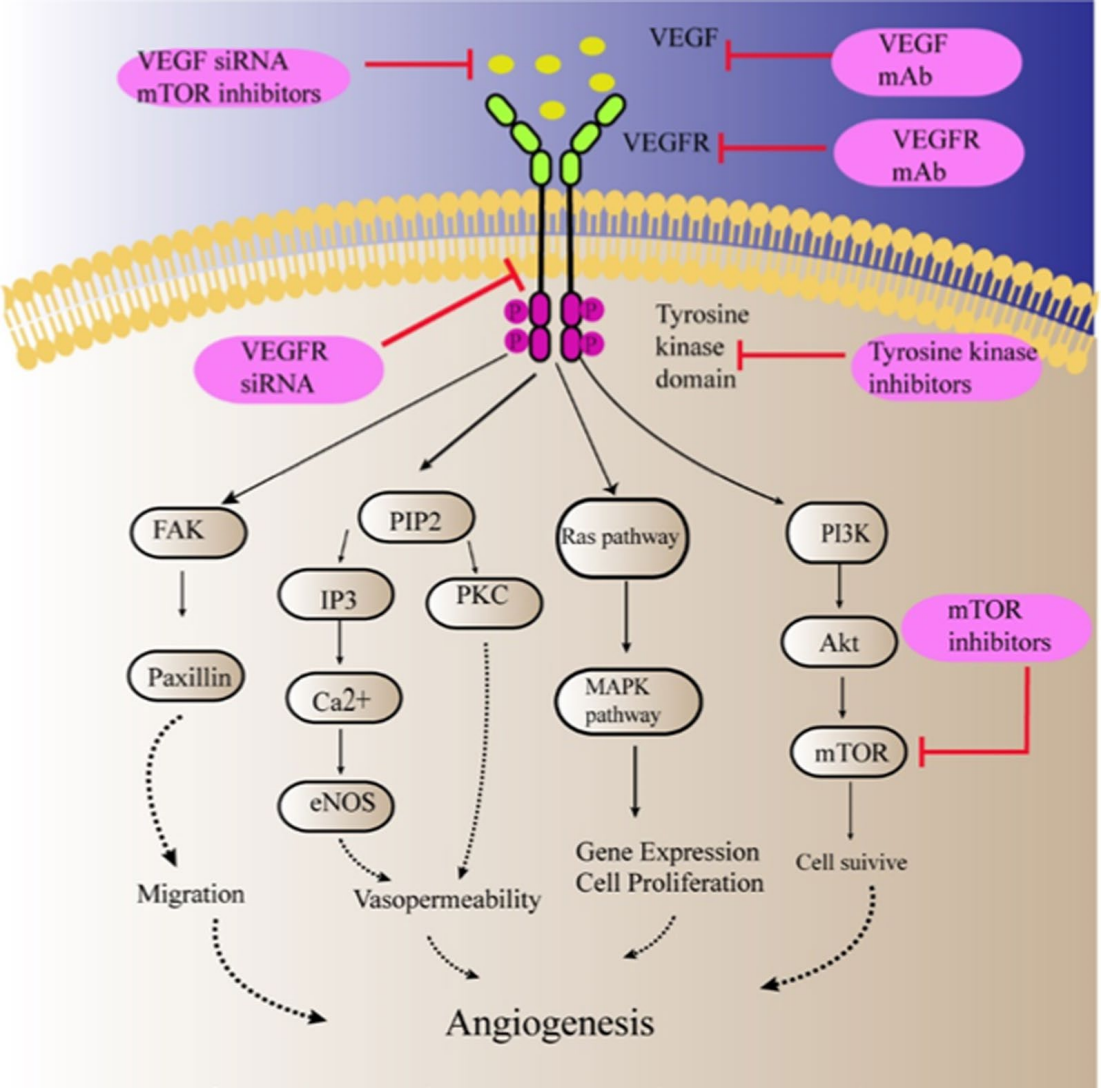
Mechanism of anti-angiogenic therapy by targeting VEGF signaling pathway

VEGF is a critical pro-angiogenic factor, which is usually overexpressed in tumor tissue. In addition to the function of promoting angiogenesis, VEGF can also promote survival and proliferation of tumor cells [9, 37, 38]. Anti-VEGF/VEGFR monoclonal antibodies (mAb), such as bevacizumab (BVZ), can block the binding of VEGF to VEGFR. Tyrosine kinase inhibitors, like vandetanib, sorafenib (SFN), could inhibit the kinase domain of VEGFR, preventing receptor activation. VEGF binding to VEGFR can activate a variety of downstream intracellular signaling pathways, including PI3K/Akt/mTOR pathway. Thus, the inhibitors of PI3K/Akt/mTOR pathways, such as rapamycin, also can block VEGF signaling pathways [5, 37, 39]. Moreover, studies have shown that rapamycin also could reduce the expression of VEGF [40]. Delivering these drugs via nanocarriers allows for better therapeutic effects with fewer side effects (Fig. 4).

Battaglia [41] prepared solid lipid NPs (SLNs) from palmitic acid and stearic acid for passive targeted delivery of BVZ. The activity of BVZ loaded in SLNs is 100 to 200 times higher than free BVZ. Wang [42] constructed a nanosystem for co-delivery of MTX and sorafenib by using the amine-terminated thermally carbonized porous silicon (PSi) as the carrier, loaded MTX on the surface and acted as the targeting ligand through chemical bonding, and loaded SFN internally via hydrophobic effect. The dual-drug co-delivery system could improve the dissolution rate of hydrophobic sorafenib, enhancing the inhibition of tumor angiogenesis. Furthermore, it also could promote the cellular uptake of MTX and achieve sustained release, enhancing the ability to kill tumor cells. Wang [43] synthesized PEG-PLA amphiphilic block copolymer self-assembled NPs, which was used to load vandetanib internally via hydrophobic effect and modify a tumor-targeting peptide iRGD on the surface. The NPs could enhance the targeting and therapeutic efficacy of vandetanib. Li [44] prepared lipid-polymer NPs based on poly (lactic-co-glycolic acid) (PLGA) to load rapamycin via hydrophobic effect and realize sustained release. The NPs could enhance the anti-angiogenic efficacy of rapamycin, and reduce the side effects and frequency of administration. Moreover, by modifying anti-VEGFR2 antibodies on the surface of NPs, the targeting and anti-angiogenic effects of the NMs can be enhanced.

In addition to the drugs mentioned above, inhibition of VEGF/VEGFR expression is also an effective anti-angiogenic therapy. The strategy of triggering VEGF/VEGFR gene silencing by delivering siRNA/shRNA via NPs, which can more efficiently and selectively down-regulate the expression of VEGF and VEGFR to interfere with the process of angiogenesis [45]. Kwak [46] used thiolated-glycol chitosan as the carrier to load the poly-VEGF siRNA through chemical crosslinking and electrostatic interaction to form stable NPs. The NPs displayed high accumulation in tumor tissue, and psiVEGF rapidly depolymerized to form monomers under the action of reductive intracellular components thereby contributing efficient gene silencing.

NMs can also realize the combination of targeting VEGF signaling pathway and other drugs to achieve synergistic therapeutic effect. Ding [47] developed a conjugated single-walled carbon nanotubes (SWNTs) modified with polyethylene imine (PEI), simultaneously loaded with candesartan (CD) and VEGF siRNA. CD was covalently bound to PEI, and siRNA was loaded inside by electrostatic interaction with SWNTs. CD could bind to the angiotensin type II 1 (AT1) receptor that expressed on neovascular ECs and tumor cells, acting as a targeting ligand and also could inhibit angiogenesis. The co-delivery NMs had specific tumor-targeting ability, enhanced lysosomal escape ability, and synergistic inhibition on angiogenesis. Huang [48] used PEGylated dendrigraft PLL NPs as the carrier, and modified with dtACPP on the surface, to simultaneously load DOX and shVEGF (plasmid expressing VEGF siRNA) complex via electrostatic interaction. DtACPP is an environmentally responsive activated cell-penetrating peptide which is dual-activated by the MMP2 enzyme and acidic pH in TME to restore activity and selectively deliver NMs to tumor cells. The NMs could effectively shutdown vessels and induce cell apoptosis in tumors.

### NMs target on other pathways

In addition to the VEGF signaling pathways, many other pathways are also involved in angiogenesis, such as Ang 2, EGF and other pro-angiogenic pathways [49]. Mutation of some tumor suppressor genes, like p35, also promote angiogenesis [50]. Pericytes are also associated with angiogenesis of tumor [51]. Targeting these sites also can achieve the purpose of inhibiting angiogenesis with NMs.

Ang 2/Tie2 is another critical pro-angiogenic signaling involved in tumor angiogenesis. Ang 2 pathway blockade has been confirmed to inhibit tumor angiogenesis in a variety of models, and has synergistic effects with VEFG blockade [52]. Shan [49] prepared chitosan magnetic NPs (CMNPs) containing plasmids expressing small interfering RNA for Ang 2 (Ang 2-CMNPs) via electrostatic interaction to silence Ang 2 expression in tumor tissues. Under a stable external magnetic field, the NPs showed good accumulation in tumor cells. The NPs displayed effective inhibition of tumor growth and Ang2 expression, and could induce the tumor cells apoptosis via the mitochondrial apoptotic pathway. Another approach is to block the Tie-2 receptor. Zhang [53] designed passive targeting, dual-responsive and self-assembled NPs based on amphiphilic peptide, mPEG_1000_-K(DEAP)-AAN-T4. The T4 is a small peptide inhibitor of Tie2 receptor. In TME, diethylaminopropyl isothiocyanate (DEAP) was protonated, exposing the AAN-T4 to legumain that is generally overexpressed in TME, resulting in AAN cleavage and T4 release, followed by the blockage of the Tie2 receptor. The P-T4 decreased vessel density significantly due to local blockage of Tie2 signaling.

Many studies have shown that the mutation of p53 gene is associated with angiogenesis. Cancer cells with p53 gene mutation exhibit the ability to promote angiogenesis, which is characterized by upregulation of pro-angiogenic factors and downregulation of angiogenesis inhibitors. Restoring p53 function in cancer cells also inhibits angiogenesis. Prabha [50] developed PLGA NPs for passive delivery of p53 plasmid DNA. The NPs had the ability to provide continuous release of DNA plasmids in tumor cells to maintain continuous gene transfection and decreased cell proliferation. After treatment, tumor tissue showed upregulation of endogenous angiogenic inhibitors, thrombospondin-1(TSP-1), as well as reduced density of microvessel and increased levels of cell apoptosis.

Pericytes are also associated with angiogenesis and can effectively maintain the stability of tumor vessels, making them a potential target for anti-angiogenic therapy. Guan [54] prepared PEG-PLA NPs modified with TH10 peptide, and loaded docetaxel via hydrophobic effect. TH10 peptide promoted cellular uptake of the NMs in pericytes via interacting with NG2 receptors. The NMs could accurately target vascular pericytes, and DTX could induce apoptosis of pericytes. The NMs could significantly prolong the survival of tumor mice without obvious toxic side effects, and the enhanced anti-tumor effect was associated with the decrease in pericytes and microvessels.

Due to the complexity of tumor angiogenic signaling pathways, anti-angiogenic therapies targeting only a single pathway often fail to achieve good therapeutic effects and lead to drug resistance. One reason is that cancer cells compensatively enhance the expression of other angiogenic signaling pathways [37]. Nanocarriers provide a good platform for simultaneous delivery of anti-angiogenic drugs targeting multiple signaling pathways to achieve better therapeutic outcomes and reduce drug resistance. In addition, some nanomaterials themselves also have anti-angiogenic effects. Mukherjee [55] demonstrated that AuNPs have anti-angiogenic properties. AuNPs can bind to heparin-binding growth factors, such as VEGF165 and bFGF, and inhibit their activity. Xu [56] demonstrated the chitosan NPs also have an anti-angiogenic effect, which was associated with the down-regulation of VEGFR2 expression.

## NMs-mediated tumor vascular blockade

NMs can also block tumor vessels by triggering coagulation reaction or gel phase transformation, thus reducing the oxygen and energy supply of tumor tissue and inhibiting tumor growth.

### NPs-loaded coagulation factor selectively induce tumor vascular infarction

Studies have shown that tumor ECs and tumor cells overexpress tissue factor (TF) on the cell surface, which results in a hypercoagulable state in TME [57]. TF is the first trigger to activate extrinsic coagulation cascade, causing coagulation reaction, promoting the formation of thrombosis and blocking vessels. The extracellular domain of TF (truncated TF, tTF) is powerless to trigger thrombus formation, and its coagulation induction activity can be recovered by targeting it near the phospholipid membrane [58]. The use of tumor-targeting peptides and TME-responsive peptides to deliver the tTF has a good potential to achieve selective tumor vascular infarction and avoid coagulation in the non-targeted sites, causing adverse reactions and improving safety. Bieker [59] engineered a fusion protein, tTF-NGR, NGR peptide can target the CD13 and integrin αvβ3 on tumor ECs. The tTF-NGR could cause partial or complete thrombotic infarction of tumor vessels after systemic administration. Shi [60] engineered a fusion protein, tTF-CREKA. CREKA is a tumor-targeting ligand, which can selectively bind to the fibrin–fibronectin complex, which is generally overexpressed in tumor vascular walls and tumor stroma. The tTF-CREKA could selectively trigger tumor intravascular thrombosis, reduce blood supply, thus inhibit tumor growth.

Another bioactive molecule that can initiate the clotting reaction is thrombin (Th). Systemic administration of Th has serious side effects due to its strong pro-coagulant activity. Therefore, targeted delivery of Th to tumor vessels through NPs can selectively induce tumor vessel infarction and reduce side effects. Li [61] constructed a nanorobot based on DNA origami that specifically transported and released Th into tumor vessels. Th was loaded in its inner cavity via chemical bonding. The two sides of DNA origami were functionalized with DNA aptamer-AS1411, so that the rectangular DNA origami sheet could be turned into a cylindrical DNA nanorobot. Moreover, the aptamer AS1411 could selectively bind to the nucleolin, which is highly expressed on tumor ECs, and the aptamer as a molecular trigger that mechanically opens the DNA nanorobot to expose Th, activating coagulation response at tumor site.

Similar to the vascular disruption caused by VDAs, in tumor vascular infarction, there are residual tumor cells in the tumor rim. NMs can realize the combination of vascular infarction and chemotherapeutic drugs. Li [62] prepared active targeting co-delivery NMs containing both Th and DOX synergistically to kill tumors. Li chose a polymer chitosan as carrier and modified the tumor-targeting peptide CREKA on the surface. The NMs showed a continuous release of Th in tumor tissue, selectively inducing intratumoral coagulation, depriving nutrients of tumors while accumulating DOX. DOX could kill tumor cells by intratumoral diffusion, including residual cancer cells at the periphery. Compared with vascular infarction monotherapy, the co-delivery NMs exhibited a higher therapeutic efficacy, with slower tumor growth and decreased tumor recurrence.

### Nanogels-mediated tumor vascular embolization

Transcatheter arterial embolization (TAE) is a tumor therapeutic technique in which the embolic agents are selectively injected into the target artery via a catheter under the guidance of imaging equipment in order to block the target artery. It is commonly used to treat hepatocellular carcinoma (HCC) [63]. The embolic agents need have a pair of contradictory properties: fluidity and embolization. Good fluidity can help it to spread rapidly into fine tumor arteries, but it is hard to realize embolization for a long time. In contrast, granular/solid embolic materials have good embolization, which can block tumor arteries for a long time, but the poor fluidity makes it hard to be transported to fine arteries. Environment-responsive nanogels can effectively solve this contradiction. In general, nanogels remain in a gel state and have good fluidity. In a specific environment, such as temperature, PH, nanogels undergo sol–gel phase transition which effectively causes tumor vascular embolization [64].

Poly (N-isopropylacrylamide-co-butyl methyl acrylate) (PIB) nanogels have been widely used in TAE therapy because of the excellent temperature-sensitive sol–gel phase transition properties. Li [64] prepared a novel composite temperature-sensitive nanogels, PIBI-2240, a composite dispersion of iohexol (240 mg/mL) and PIB nanogel (2.0 w/v%). In a tumor vascular simulation model, the PIBI-2240 was able to sufficiently embolize all levels of micro-channels. And the good embolism ability was further demonstrated in the normal rabbits’ renal arteries. Moreover, PIBI-2240 also could reduce the expression of VEGF, HIF-1αand CD31 in the TAE-treated tumor tissue, which resulted in effective inhibition of tumor vascular re-canalization and collateral circulation.

The margin of tumor tissue is the most likely area for residual cancer cells and recurrence after TAE. Nanogels can also achieve TAE in combination with chemotherapeutic agents (transcatheter arterial chemo-embolization, TACE). Qian [65] prepared DOX-loaded PIB nanogels–iohexol (IBi-D) dispersions. The studies showed that IBi-D dispersions had good embolization ability to renal arteries of rabbits at all levels by controlling injecting dosages. IBi-D dispersions could also sustainedly release DOX while embolizing tumor vessels, achieving better therapeutic effects. Nanogels can also be used in TAE combination with anti-angiogenic drugs. Zhou [66] used PIB nanogels to load apatinib, which is an inhibitor of VEGFR2. PIB nanogels could effectively deliver apatinib to tumor tissue, and achieve sustained release. While embolizing tumor vessels, the nanogels continuously released apatinib in the tumor margins and synergistically inhibited tumor growth.

## NMs-mediated tumor vascular normalization

The abnormal tumor vessels are recognized as an important culprit of failure in tumor therapy. Tumor vascular normalization through correcting the abnormal state of tumor vessels, may promote tumor vessels mature, restore the normal function of the tumor vessels, increase perfusion and oxygen levels, and elevate the transport and efficacy of anti-tumor drugs [2]. NMs as ideal vehicle with flexible modification of bio-materials can better normalize tumor vessels and fulfill combined delivery.

### Anti-angiogenic NMs-mediated tumor vascular normalization

The main reason for the abnormality of tumor vascular structure and function is that the overexpression of pro-angiogenic factors breaks the balance between the anti-angiogenic signals and pro-angiogenic signals in tumor tissue. When properly used, low doses of angiogenesis inhibitors can restore this balance, appropriately reversing tumor vessels to a more mature phenotype, enhancing perfusion and oxygenation, while promoting the transport of chemotherapeutic drugs [2, 11, 67].

The Blocking of VEGF signaling pathway by NMs can normalize tumor vessels. Cho [68] used a novel, SS cleavable, pH-activated lipid-like material to form NPs and modify RGD peptide on the surface to deliver VEGFR2 siRNA actively to tumor ECs. The NPs can be degraded in response to specific intracellular environments, releasing VEGFR2 siRNA. The NPs can effectively down-regulate VEGFR2 expression in tumor ECs, which normalized tumor vessels. Clavreul [69] prepared lipid nanocapsules (SFN-LNCs) to encapsulate SFN via hydrophobic effect. Compared with free SFN, the NMs were more effective in inducing glioblastoma vascular normalization, which was manifested as decreased tumor vessel area and increased tumor blood flow. SFN-LNCs could improve the effect of chemotherapy and radiotherapy in glioblastoma by inducing vascular normalization. Ang2/Tie2 signaling pathway is also an important contributor to the increase of tumor vascular permeability and further vascular instability. Blocking of Ang2 signaling pathway can also normalize tumor vessels. Increased vascular regression to normal phenotype in TME was also observed in Ang2-CMNP mediated Ang 2 silencing [49].

Other anti-angiogenic NMs can also normalize tumor vessels. Endostatin is an endogenous angiogenesis inhibitor which interrupts VEGF-induced angiogenesis. Recombinant human endostatin (rhES) is a modified endostatin with better stability. Li [70] constructed AuNPs loaded with rhES to target tumor tissues via EPR effect for short-term treatment. The NMs could lead to transient normalization of tumor blood vessels, which is manifested as enhanced vascular integrity, increased blood perfusion, decreased vascular permeability and alleviated hypoxia, thereby increasing drugs delivery and improving therapeutic efficacy. Anti-angiogenic therapy mediated by AuNPs can also be achieved vessel normalization. Huang [71] proved AuNPs could strengthen tight junctions of ECs and increase pericyte coverage, which restored vascular integrity and normalized tumor vessels. Moreover, AuNPs-induced vascular normalization promoted the accumulation of CD3^+^CD8^+^ T cells in TME, which enhanced the response to anti-PD-L1 immunotherapy.

### Other NMs-mediated tumor vascular normalization

In addition to anti-angiogenic drugs, there are other molecules or methods that normalize tumor vessels.

Dopamine (DA) can normalize tumor vessels by acting on DA receptors on vessels. DA can up-regulate angiopoietin-1 (Ang1, a factor that can promote vascular pericyte recruitment and vascular maturation) in pericytes, and also can inhibit VEGF. Taleb [72] used MSNs with amine groups (NH_2_) as the carrier, and modified with phenylboronic acid (PBA) by conjugation. and DA was loaded into NPs by reacting with PBA. Because the boronic-ester bond between DA and PBA is hydrolyzed in the weakly acidic TME, the NMs resulted in stable delivery and local release of DA. The vascular normalization by MSNs@DA resulted in decreased vascular leakiness and permeability, as well as increased delivery, penetration and anti-tumor activity of chemotherapeutic drugs.

NO also plays a certain part in regulating angiogenesis and maintaining vascular homeostasis, which can induce normalization of tumor vessels. Sung [73] synthesized a Nano-NO delivery system, PEG modified PLGA NPs internally loaded with dinitrosyl iron complex (DNIC), to passively target tumors through EPR effect. The biodegradable PLGA polymer offered a sustained release of NO from DNIC, which is the NO donor in the nanosystem. Low-dose Nano-NO therapy could induce tumor vascular normalization and enhance the delivery and efficacy of anti-cancer drugs. Furthermore, low-dose Nano-NO reversed the immunosuppressive TME into the immunosupportive type, which improved the effect of immunotherapy.

Metronomic chemotherapy (MET) refers to continuous low dose, high frequency administration of chemotherapeutic agents. MET also can act on ECs, inhibit cell proliferation and induce apoptosis, and show anti-angiogenic effect. NMs-mediated MET can improve tumor specific biodistribution and reduce side effects. Luan [74] synthesized paclitaxel-loaded NPs (F56-PTX-NP) with surface modified F56 peptide from PEG-PLA. F56 peptide is a ligand that could selectively bind to the VEGFR-1 on ECs. Metronomic NPs could effectively target ECs, cut off excess immature vessels through anti-angiogenic activity, and induce ECs to secrete TSP-1, an endogenous angiogenesis inhibitor. The treatment could normalize tumor vessels, characterized by the coverage of BM and pericytes increased significantly, IFP decreased, pO2 increased, perfusion and DOX delivery also increased.

### Combination with chemotherapy

Tumor vascular normalization is a short process in therapy, and excess or prolonged therapy will lead to further vascular disruption. Therefore, enhancing delivery and efficacy of chemotherapeutic drug through vascular normalization needs to be completed within a specific window period. However, due to the lack of effective markers indicating the therapeutic window of vascular normalization, it is difficult to accurately grasp the specific window period to enhance anti-tumor therapy [9, 75]. Co-delivery of vascular-normalizing drugs and chemotherapeutic drugs via NMs to simplify and enhance vascular normalization strategy may be a solution.

Du [76] synthesized a novel lipid derivative conjugate NM from cholesterol, which contain both low molecular heparin (LMWH) and the chemotherapeutic drug gemcitabine via chemical bonding. LMWH is an angiogenesis inhibitor and can block the VEGF signaling pathway. This NMs utilize the synergistic effect of anti-VEGF therapy with MET to promote the normalization of tumor vessels. What ‘s more, the NMs have the ability to further load cytotoxic drugs, such as PTX, to form a "nano-community" that normalized tumor vessels while delivering the loaded PTX, effectively enhancing the efficacy of PTX without the need to determine the exact time of the effective therapeutic window.

NMs also can achieve multi-strategy synergistic therapy. Li [77] prepared an anionic liposome NMs with a sandwich structure for vascular normalization in combination with chemotherapy and sonodynamic therapy. Indocyanine green (ICG), an ultrasonic sensitizer, was encapsulated in the external hydrophobic region, while topotecan (TPT), a chemotherapeutic agent, was loaded in the internal hydrophilic region, and the erlotinib (ERL)a vascular-normalizing drug with positively charged was loaded in the outermost layer of anionic NPs via electrostatic interaction. The NMs entered tumor through ERL-induced vascular normalization. Ultrasound treatment mediated by ICG induced a temporary induction of vascular permeability, inhibited angiogenesis, induced vascular normalization in conjunction with ERL, and promoted the accumulation of TPT in tumors. Furthermore, TPT not only had cytotoxic effects, but also prolonged the duration of vascular normalization by reducing HIF-1α expression. The nanosystem also effectively improved the TME and enhanced immune response and prognosis.

## NMs target tumor vessels to enhance immunotherapy

Tumor immunotherapy is intended to activate and enhance the immune system's ability to specially identify and remove cancer cells, including cancer vaccine, immune checkpoint therapy, adoptive T cell therapy [78]. Immunotherapy has greatly expanded anti-tumor therapy, raising hopes of eradicating tumors. Immune checkpoint blockade (ICB), such as PD-1/PD-L1 blockage, have displayed long-lasting and significant therapeutic effects in a variety of cancers [79]. However, there is still a significant proportion of patients who do not respond to ICB and benefit from it [80]. This condition may be attributed to the impaired infiltration of anti-tumor immune cells in tumor and the immunosuppressive TME caused by abnormal tumor vessels. Therefore, it may be a promising method to act on tumor vessels with NMs to increase the accumulation of anti-tumor immune cells, reverse the immunosuppressive TME, thus improve the therapeutic effect of immunotherapy.

### NMs normalize the immunosuppressive TME through vascular normalization

Tumor vascular normalization is often accompanied by increased T cell infiltration and the improvement of the immunosuppressive TME. Cho [68] proved that VEGFR2 knockdown mediated vascular normalization markedly increased CD8^+^ T cell infiltration in TME. Compared with PD-1 mAb alone, the combination therapy was more effective in inhibiting tumor growth and improving the response rate to PD-1 mAb. Huang [21] demonstrate that targeting tumor vessel with low anti-VEGFR2 mAb induced in vascular normalization, which was manifested in more uniform distribution of functional vessels, and reversed immunosuppressed TME, as evidenced by promoting the polarization of TAM from M2 phenotype towards M1 phenotype and increasing the tumor accumulation of CD4^+^and CD8^+^ T cells. Improvement of immunosuppressive TME was also observed in vascular normalization induced by Nano-NO mentioned above [73]. Low-dose Nano-NO could improve infiltration of T cells in HCC, induce down-regulation of PD-L1 expression, inhibit the polarization of TAMs to M2 phenotype, which means the reversal of TME. The vascular normalization induced by Nano-NO could effectively enhance the therapeutic effects of cancer vaccines.

On the one hand, vascular normalization restores the normal function of blood vessels, increases perfusion, improves oxygenation of tumor tissue, relives hypoxia. Improved vascular function can restore the ability of tumor vessels to transport immune effector cells into TME, and the increased oxygen levels can reduce immunosuppression induced by hypoxia. On the other hand, vascular normalization reduces the level of pro-angiogenic factors, like VEGF, and relieves immunosuppression induced by them. Vascular normalization increases anti-tumor immune cell infiltration and function, transforming immunosuppressive TME into an immunosupportive phenotype. Moreover, studies have shown that there is a mutually reinforcing process between vascular normalization and immune activation. Tian [81] reported that the inactivation or depletion of CD4^+^ T cells could disrupt vascular normalization, reduce vascular pericyte coverage, and increase metastasis. The ICB could activate the type 1 helper T cells, which secrete INF-γ, to promote the normalization of tumor vessels. Zheng [82] reported that ICB promoted the accumulation of CD8^+^ T cell and the production of IFN-γ, leading to vascular normalization, as shown by increased perfusion. Tumor immune reprogramming and vascular normalization are mutually reinforcing processes. It may be an ideal approach to treat tumors with NPs loaded with both anti-angiogenic drugs and immune checkpoint inhibitors, which can complement each other to achieve better therapeutic outcomes.

### NMs-mediated platelet inhibition

Platelets play a vital role in protecting tumor vascular integrity and maintaining vascular barrier, which limits the transport and efficacy of chemotherapeutic agents. Local depletion of tumor-associated platelets has been suggested to enhance vascular permeability, thereby improving the efficacy of chemotherapeutic drugs [83]. Servais [84] confirmed that platelet inhibition also promoted the T cells infiltration in TME, thus restoring the anti-tumor immune response. Selective inhibition of tumor platelets through NMs to destroy tumor vascular barrier and enhance anti-tumor immune cells infiltration is also a method of enhancing immunotherapy.

Zhou [85] prepared albumin-based perfluorotributylamine NPs (PFTBA@Alb) that could selectively inhibit tumor platelets and enhance vascular permeability. PFTBA@Alb could enhance the accumulation of CD4^+^ and CD8^+^T cells in TME. More importantly, through combined therapy with anti-PD-L1 antibodies, tumor inhibition rate was significantly increased to nearly 90%. NO is an endogenous platelet inhibitor, which can inhibit various functions of platelets. Xu [86] used albumin as a carrier, surface modified NO donor (S-nitrosothiols, SNO), and loaded with chemotherapeutic drug PTX, to prepare an ultrasonic-responsive nanosystem. Under the action of ultrasound, the nanosystem could release NO, which can break the tumor vascular barrier and promote the accumulation of PTX and infiltration of T cells. Moreover, due to the improved vascular permeability, oxygen-carrying hemoglobin could penetrate into tumor tissue and reduce hypoxia, which is beneficial for immunotherapy and chemotherapy.

### NMs-mediated blockade of immune checkpoint on ECs

Tumor tissue restricts T cell function and induces T cell apoptosis through overexpression of immune checkpoint (PD-L1, CTLA-4) [87]. NPs loaded with immune checkpoint inhibitors can more effectively block their function and restore function of T cell.

Using iron-dextran NPs as the carrier, Kosmides [88] synthesized an “immunoswitch” NPs to overcome the immunosuppressive TME. Two different antibodies, PD-L1 inhibitory antibody and 4-1BB agonist antibody, are simultaneously loaded on the surface of NPs. The NPs could block the PD-L1 pathway to eliminate the apoptosis or inactivation of CTLs induced by PD-L1, and simultaneously activate the co-stimulatory 4-1BB pathway on CTLs to enhance cytotoxicity of CTLs. The immunoswitch NPs could effectively increase the accumulation of CTLs in TME and enhance their specificity and ability to remove cancer cells. Tumor ECs also express PD-L1 on the surface, so theoretically, this NPs can also act on tumor ECs to promote CTLs infiltration. Moreover, Asís [89] provided evidence that 4-1BB is also is also present on the surface of tumor ECs. Agonist mAb stimulated tumor ECs, could increase expression of the ICAM-1, VCAM-1, and E-selectin on the surface of ECs, which are essential for CTLs to cross vessels and transport into TME. The stimulation of 4-1BB not only enhanced function of T cells, but also increased the ability of T cells to infiltrate into tumor tissue by acting on tumor ECs. Thus, in theory, the “immunoswitch” NPs can also act on ECs to up-regulate the level of adhesion molecules on cell surface, and enhance tumor infiltration and anti-tumor ability of CTLs.

## Conclusion and outlook

Solid tumors are malignant and refractory, to some extent, attributing to the structural and functional abnormalities of tumor vessels, and the hypoxic, acidic and immunosuppressive TME, which impair the therapeutic efficacy of anti-tumor drugs. Vessel-targeting strategy provides a new idea and target for anti-tumor therapy. Three nanotechnology-based anti-vascular strategies, vascular disruption, anti-angiogenesis and vascular blockade, show enhanced anti-tumor effects by disrupting vascular function rather than directly killing tumor cells. In addition to obstructing vascular function, it is also a promising method to improve the efficacy of chemotherapy, radiotherapy and immunotherapy by correcting abnormal tumor vessels and TME, thereby promoting the delivery of anti-tumor drugs and immune cells to tumor tissue.

However, single destructive vessel-targeting strategy often fails to achieve sufficient therapeutic efficacy, often accompanied by drug resistance or tumor recurrence. The combined therapy strategy based on nanotechnology can not only co-deliver different vascular targeting drugs to maximize the effect of “starve tumor”, but also combine anti-vascular therapy with chemotherapy and radiotherapy to synergize the anti-tumor effect. In addition, nanotechnology-based vascular normalization combined with anti-tumor therapy, as well as multi-strategy synergistic therapy, provide a potential method to overcome the shortcomings of traditional anti-tumor therapy and simplify the combined therapy. Moreover, due to the mutual promotion between vascular normalization and immune reprogramming, co-delivery of vascular-normalizing drugs and immune checkpoint inhibitors in one nanoplatform may improve tumor immunotherapy to a greater extent. Nanotechnology-based multi-strategy synergistic therapy may be a promising strategy for future vessel-targeting therapy.

Although NMs show great promise in tumor vessel-targeting therapy. Most of the vessel-targeting NMs mentioned in this review are still in the laboratory research stage, and there are still some issues to be considered. The first is the selection of more specific targeting ligands and response factors, which should be selected to avoid accumulation or early release in non-tumor sites. Secondly, the biocompatible nanomaterials are also an important factor determining the application prospect of NMs. Biodegradable protein, polymer nanocarriers and biomimetic NPs wrapped in cell membranes both have good biocompatibility and great application potential [90]. In addition, inorganic or metal nanomaterials have unique advantages in tumor diagnosis and treatment due to their unique properties, which is also an important development direction in the future. For example, the photothermal effect and anti-angiogenesis of Au NPs, and the anti-tumor effect of arsenene nanodots and surface-oxidized arsenene nanosheets [91, 92]. Finally, the development of multifunctional nanocarriers with simple design is also a major challenge of NMs. The unification of diagnosis, treatment, imaging and other functions into a nanocarrier and the realization of real-time visualization of the treatment process will greatly improve nanotherapeutics, but the design should not be too complicated, otherwise it will be not conducive to large-scale production and clinical treatment [93]. The development of nanomaterials with good bio-compatibility, safety, no side effects, precise targeting, simple design and multi-functional combination will open new horizons for nanotherapeutics.

## Data Availability

Not applicable.
